# The feasibility of pelvic floor training to treat urinary incontinence in women with breast cancer: a telehealth intervention trial

**DOI:** 10.1007/s12282-022-01405-6

**Published:** 2022-09-26

**Authors:** Udari N. Colombage, Sze-Ee Soh, Kuan-Yin Lin, Jennifer Kruger, Helena C. Frawley

**Affiliations:** 1 Department of Physiotherapy, Faculty of Medicine, Dentistry and Health Sciences, Melbourne School of Health Sciences, The University of Melbourne, 161 Barry St, Carlton, VIC 3053 Australia; 2 Institute of Health and Wellbeing, Federation University, Churchill, VIC Australia; 3 Department of Physiotherapy, Monash University, Melbourne, VIC Australia; 4 Department of Epidemiology and Preventive Medicine, Monash University, Melbourne, VIC Australia; 5 Department of Physical Therapy, National Cheng Kung University, Tainan, Taiwan; 6 Institute of Allied Health Sciences, College of Medicine, National Cheng Kung University, Tainan, Taiwan; 7 Auckland Bioengineering Institute, The University of Auckland, Auckland, New Zealand; 8 Junofem, Auckland, New Zealand; 9 Allied Health Research, Mercy Hospital for Women, Melbourne, VIC Australia; 10 Allied Health Research, Royal Women’s Hospital, Melbourne, VIC Australia

**Keywords:** Breast cancer, Urinary incontinence, Pelvic floor muscle training, Telehealth

## Abstract

**Purpose:**

To investigate the feasibility of recruiting into a pelvic floor muscle training (PFMT) program delivered via telehealth to treat urinary incontinence (UI) in women with breast cancer on aromatase inhibitors.

**Methods:**

We conducted a pre-post single cohort clinical trial with 54 women with breast cancer. Participants underwent a 12-week PFMT program using an intra-vaginal pressure biofeedback device: femfit^®^. The intervention included eight supervised individual PFMT sessions over Zoom^™^ and a 12-week home exercise program. The primary outcome of this study was feasibility, specifically consent rate. Secondary outcomes which included prevalence and burden of UI measured using the International Consultation on Incontinence Questionnaire–Urinary Incontinence Short Form (ICIQ-UI SF), and pelvic floor muscle (PFM) strength measured as intravaginal squeeze pressure were compared using McNemar’s and paired *t* tests.

**Results:**

The mean age of participants was 50 years (SD ± 7.3). All women who were eligible to participate in this study consented (*n* = 55/55, 100%). All participants reported that the program was beneficial and tailored to their needs. The results showed a statistically significant decline in the prevalence (percentage difference 42%, 95% CI 28, 57%) and burden (ICIQ-UI SF score mean change 9.4, 95% CI 8.5, 10.4) of UI post intervention. A significant increase in PFM strength was observed post-intervention (mean change 4.8 mmHg, 95% CI 3.9, 5.5).

**Conclusion:**

This study indicated that PFMT delivered via telehealth may be feasible and potentially beneficial in treating stress UI in women with breast cancer. Further studies such as randomized controlled trials are required to confirm these results.

## Introduction

Pelvic floor muscle training (PFMT) is the recommended first-line management of urinary incontinence (UI) [1]. Despite the high prevalence of UI (38%), specifically stress UI (37%), in women with breast cancer compared to women without breast cancer (21%) [2], PFMT to treat UI does not appear in breast cancer care pathways [3]. The potential link between breast cancer and UI is presumed to be a result of ovarian suppression secondary to the use of endocrine therapy [4]. Aromatase inhibitors (AIs) are a type of endocrine therapy prescribed to women with breast cancer, which diminish estrogen synthesis and are known to have adverse effects on the function of pelvic floor (PF) tissues [5]. There is some evidence that women with breast cancer taking AIs have weak PF muscle strength and endurance, and that the long-term use of AIs is negatively associated with PF muscle endurance [6]. If PF muscle strength can be improved, this may compensate for impairments in other PF tissues, as well as directly improving the function of the PF muscles [7, 32]. Therefore, there may be a role for PF conservative therapies, such as PFMT to treat stress UI in women following treatment for breast cancer.

While a recent study [8] recommended PFMT and bladder training in the management of genito-urinary symptoms in women with early breast cancer on endocrine therapy, this recommendation was not underpinned by results from a clinical trial in women with breast cancer. No trials of PF therapies have been conducted to test the effectiveness of PFMT in women with breast cancer to date. Due to the potential effects of cancer treatments on PF structures, women with breast cancer may respond differently to PF therapies compared to women without breast cancer. Therefore, population-specific trials (phase II) are required to understand the feasibility and safety of PFMT in women with breast cancer before an effectiveness study can be conducted.

The COVID-19 pandemic has meant that physiotherapists have had to explore innovative ways to deliver PFMT remotely via telehealth [9]. Telehealth involves providing healthcare remotely via digital communication technology such as Zoom [10]. The correct contraction of PF muscles is required to increase PF muscle strength, and therefore the success of PFMT [11]. While PFMT can be instructed via telehealth [9], without a face-to-face clinical examination, a physiotherapist is unable to assess and confirm if the patient is able to perform a correct PF muscle contraction. A strategy to minimize this limitation is for the patient to use a self-monitoring intra-vaginal device such as the femfit^®^ [12], which provides real time information to patients and researchers about a participant’s PF muscle contraction technique during each telehealth session.

A systematic review comparing telehealth and face-to-face PFMT found that interventions delivered via telehealth may improve participant adherence to PFMT [9]. However, as these studies were conducted in women without cancer, we do not know if similar results will be observed in women undergoing treatment for breast cancer. Additionally, none of the studies in this review provided individualized physiotherapy sessions with the use of a biofeedback device for PFMT [9].Therefore, a phase II pre-post single cohort trial was conducted to assess the feasibility of recruiting into telehealth-delivered PFMT program as an intervention to treat stress UI in women with breast cancer. We hypothesized that PFMT would be a safe and feasible therapy option for women with breast cancer and stress UI.

## Methods

This study is reported according to the Standard Protocol Items: Recommendations for Interventional Trials (SPIRIT) statement [13], and the Consensus on Exercise Reporting Template (CERT)-PFMT variation [14]. Ethics approval was obtained from Human Research Ethics Committee (ID: 2021-22456-23099-5).

### Trial design

This phase II pre-post single cohort pilot clinical trial investigated the feasibility of telehealth-delivered PFMT as an intervention to treat stress UI in women with breast cancer. This trial was registered with the Australian New Zealand Clinical Trials Registry (registration number: ACTRN12621000794808).

### Study setting

The study was conducted in Australia. The telehealth sessions were conducted via a web-based platform (Zoom^™^).

### Eligibility criteria

We included women (≥ 18 years old) who had been diagnosed with breast cancer (stages I–IV), and were receiving aromatase inhibitors for cancer treatment and experiencing stress UI (urinary leakage with cough or sneeze) at the time of enrolment. All participants were required to have access to a mobile device with internet access and Zoom^™^. Women who were unable to communicate in English were excluded.

### Intervention

**Fig. 1:**
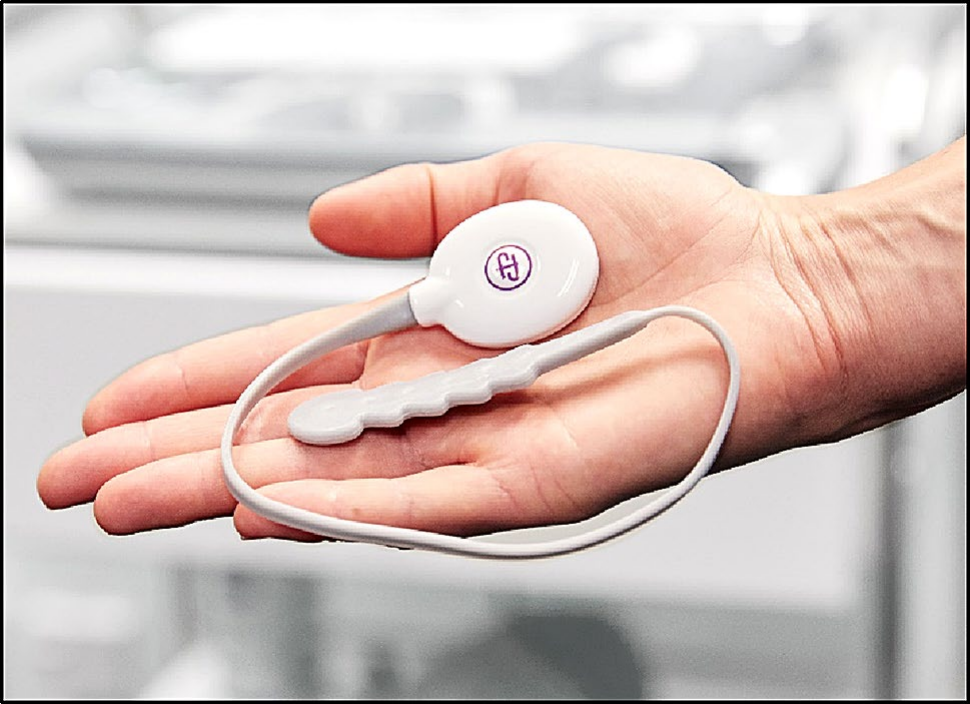
Intra-vaginal pressure biofeedback device, femfit^®^. Image used with permission from Junofem

Participants underwent a 12-week PFMT program using an intra-vaginal pressure biofeedback device—femfit^®^ (see Fig. 1). All sessions of the program were conducted by a female researcher (UC), a registered physiotherapist, who had undergone postgraduate PF physiotherapy training to enable her to deliver this intervention. The exercise program was delivered via Zoom^™^. All participants joined each session from their home.

**Fig. 2:**
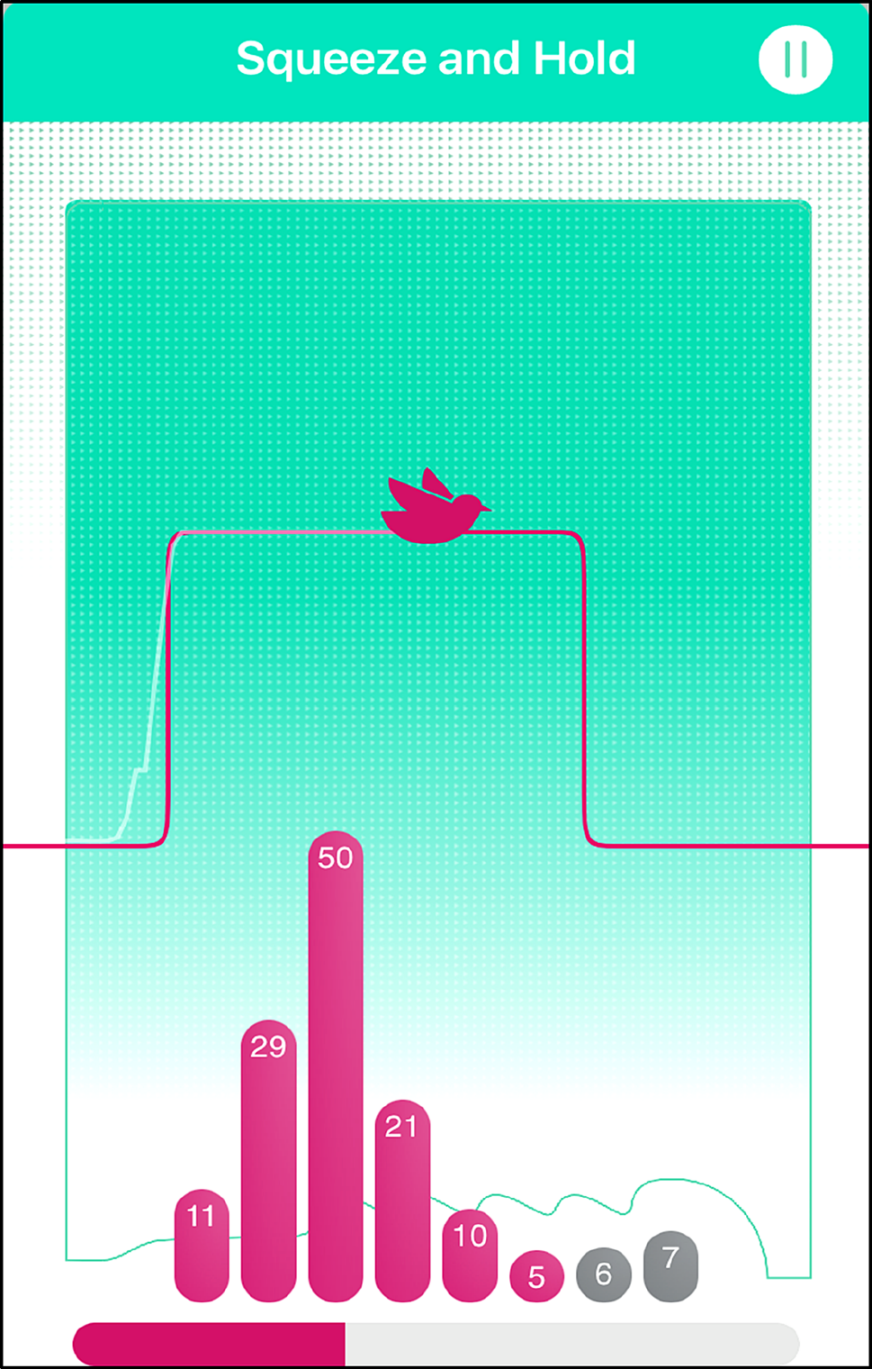
Femfit^®^ phone application. Image used with permission from Junofem

In the first telehealth session, participants learnt how to contract their PF muscles, how to use the femfit^®^ device, and how to complete their home exercise program. The pressure readings from the femfit^®^ were displayed via an app on their mobile phone (see Fig. 2). The display which consisted of eight bars each corresponding to a pressure sensor on the femfit device, was used to indicate when the participant was able to correctly contract their PF muscles. For example, co-contraction of abdominal muscles was indicated by high abdominal pressure readings, reflected by bars seven and eight (in grey) being at the same level as the bars which showed pelvic floor activation. This was then corrected by the researcher through verbal cues.

Participants received eight supervised, individual PFMT sessions via Zoom^™^ and 12 weekly check-ins with the physiotherapist via email. They followed a home exercise program installed on the femfit^®^ phone application which was based on a published PFMT program [15]. The intra-vaginal sensor was only used during the supervised sessions to confirm PF muscle contraction technique. When the femfit^®^ device was not used, the femfit^®^ phone application guided the PF home exercise program using visual cues.

Participants completed three sets of six to ten maximal contractions, six to ten fast contractions, three podium (endurance) contractions and three knack contractions per PFMT session [15]. The number of repetitions and duration of each contraction increased as the program progressed. Participants were instructed to complete the PFMT program five times per week. The program progressed every 4 weeks by either increasing the duration of each contraction or increasing the load of the exercise by progressing from across-gravity (lying) to against-gravity (sit or stand) positions. The program was tailored to each individual by varying the home exercise program progression earlier or later than the set 4-week interval.

Participant adherence to the exercise program was monitored through an exercise diary incorporated into the femfit^®^ phone application which acted as a motivation strategy. Other motivation strategies included weekly check-ins with the physiotherapist which covered education on how PFMT may help their symptoms, setting short term goals, exploring enablers and barriers to completing their exercises and setting reminder notifications from the femfit^®^ phone application to complete their home exercise program [16].

### Recruitment

The study flyer containing the participant information sheet and a link to an e-questionnaire was shared on various social media pages that connect with women with breast cancer including the Breast Cancer Network Australia. Women who expressed their interest in participating in the study through the e-questionnaire and completed the e-consent form were contacted by a researcher to confirm eligibility and schedule their first telehealth session. Participants completed the pre-intervention questionnaire and received the femfit^®^ device via post prior to the first telehealth session. After the first session, participants had weekly follow-up sessions for the first month, and fortnightly follow-up sessions for the second and third months. Participants completed the post-intervention questionnaire after the final session in week 12.

### Outcomes

#### Sociodemographic and medical outcomes

Sociodemographic and medical outcomes including age, height, weight, parity, postcode, home situation, relationship situation, educational level, employment status, smoking status, medical history, and cancer history were collected via the e-questionnaire prior to starting the intervention. Postcodes were used to classify participants living in urban and country areas of Australia according to the Australian Statistical Geography Standard (ASGS). Section of State categories 0 and 1 were classified as urban while categories 2 and 3 were classified as country [17].

#### Feasibility outcomes

The feasibility outcomes of this study included [18]:

- Consent rate: consent rate was the primary outcome. We chose consent rate as the primary outcome as we wanted to know whether women would be interested in participating in a PFMT program as per the guidelines recommended by the National Institute for Health and Care Research which advises that willingness to participate in an intervention should be established [19, 20]. This was calculated at the end of the recruitment period using the number of participants who consented for the study divided by the total number of participants who were eligible but did not consent to the trial.
- Retention rate: retention rate was calculated at the end of the intervention period using the number of participants who complete the 12-week trial divided by the number of participants who initially consented to the trial.
- Attendance rate: attendance rate was calculated at the end of the intervention period using the number telehealth of sessions attended out of eight.
- Adherence rate: the adherence to home exercise program was calculated at the end of the intervention period using the number of days the participant completed the home exercise program divided by 83 days (12 weeks).
- Withdrawal rate: the withdrawal rate was calculated at the end of the intervention period using the number of participants who withdrew from the trial after consenting divided by the number of participants who initially consented to the trial.
- Adverse and serious adverse events: the number of adverse or serious adverse events was assessed during telehealth consultations throughout the 12-week intervention period. Participants were asked about any pelvic symptoms (such as pain, intravaginal bleeding or itching) at each session. They were also encouraged to contact the research team as soon as they experienced any of these symptoms.
- Acceptability: the acceptability of the intervention was assessed using purpose-designed questions related to the participant experience of PFMT exercise program and use of femfit^®^ device and phone application.
- Satisfaction: the satisfaction of the intervention was assessed using a five-point Likert-scale ranging from ‘1 = very dissatisfied’ to ‘5 = very satisfied’.

#### Clinical outcomes

The clinical outcomes were:

- Pelvic floor muscle strength: this was assessed by measuring the intravaginal squeeze pressure using the femfit^®^ device. This outcome was recorded from the first and last supervised telehealth sessions. Pelvic floor muscle strength measurements were extracted from the PFMT program on the femfit^®^ phone application which had different programs at week 1 (six second hold squeezes) and week 12 (ten second hold squeezes). The mean of the first three maximum PF activation pressures was used as the PF muscle strength score in mmHg. Intra-abdominal pressure measured by sensor 8 of the femfit^®^ device was subtracted from PF pressure measured by sensors 3–6 of the femfit^®^ device to obtain the isolated PF muscle activation pressure [21]. The femfit^®^ device has been shown to have excellent test–retest reliability in measuring intravaginal squeeze pressure during PF muscle contraction in supine and standing positions [22].
- Prevalence, frequency, severity and impact of UI: this was assessed using the International Consultation on Incontinence Questionnaire-Urinary Incontinence Short Form (ICIQ-UI SF) [23] before and after the intervention. The ICIQ-UI SF [23] is a self-administered instrument which has been validated for use in women with UI [23]. It consists of six items. Item three assesses the frequency of UI according to the response options as follows: 0 = never, 1 = about once a week or less often, 2 = two or three times a week, 3 = about once a day, 4 = several times a day and 5 = all the time. Item four assesses the severity of UI (the amount of leakage) according to the response options as follows: 0 = none, 2 = a small amount, 4 = a moderate amount, and 6 = a large amount. Item five assess the impact of UI on participants’ quality of life on a 11-point Likert scale with 0 = no impact at all to 10 = a great deal of impact. Scores for items three, four and five are added to obtain the scale score ranging from 0–21 with higher scores indicating a higher impairment of UI [23]. Item six is a self-diagnostic item and is not scored. The ICIQ-UI SF has been shown to have good construct validity, convergent validity and test–retest reliability [23].
- Participant impression of change in UI: this was measured using the Patient’s Global Impression of Change (PGIC) scale [24] at the completion of the intervention. This scale measures the participant’s perceived change in symptoms. It consists of one item which asks participants to rate their impression of change in UI from pre- to post-PFMT intervention (response options: 1 = no change or worse, 2 = almost the same, 3 = a little better, 4 = somewhat better, 5 = moderately better, 6 = Better, 7 = a great deal better). The scale score ranges from 1 to 7 with higher scores indicating greater improvement in UI [24]. The questionnaire has been shown to have good concurrent validity in women with pelvic organ prolapse [25] and has been used in many studies with women with UI to assess participant impression of change in UI symptoms [26, 33].

### Sample size

The primary outcome of this study was feasibility, specifically consent rate. The sample size calculation was based on an estimated consent rate of 15% which was slightly lower than that observed in a previous similar study by the research team (17%) which recruited women with breast cancer from a clinic [27]. Setting precision at 0.1 and an anticipated consent rate of 15%, 49 participants in total was estimated to be sufficient to demonstrate the feasibility of the trial. Allowing 10% for attrition, we recruited a total of 54 participants for this study [20].

### Statistical methods

Participant demographics and summary scores from questionnaires were reported descriptively. Feasibility data were also reported using descriptive statistics. Pre- and post-intervention measures of PF muscle strength scores from the femfit^®^ and the ICIQ-UI SF [23] were compared using paired t-tests for continuous variables, and McNemar’s test for categorical variables. All analyses were conducted using Stata v16.0/IC (StataCorp, LLC).

## Results

### Participant characteristics

Table 1 presents the demographic and clinical characteristics of 54 participants. The mean age was 50 years (SD ± 7.3). Participants living in both urban (*n* = 33/54, 61%) and country (*n* = 21/54, 39%) areas of Australia according to the ASGS [17] participated in this study.

**Table 1:** Participant characteristics

Variables	Women with breast cancer (*n* = 54)
Age, years mean (SD)	50.2 (7.3)
Body mass index, kg/m^2^ mean (SD)	28.0 (6.7)
Parity, mean (SD)	2.1 (0.9)
Menopausal status, *n* (%)	
Menstruating	0 (0)
Peri-menopausal	3 (6)
Post-menopausal	51 (94)
Home situation, *n* (%)	
Home alone	12 (22)
Home with others	42 (78)
Rurality, *n* (%)	
Urban area	33 (61)
Country area	21 (39)
Relationship situation, *n* (%)	
Single	13 (24)
In a relationship/married	41 (76)
Educational level, *n* (%)	
High school or less	13 (24)
University	41 (76)
Employment status, *n* (%)	
Not working	20 (37)
Working	34 (63)
Breast cancer stage at diagnosis, *n* (%)	
Stage I	11 (20)
Stage II	24 (44)
Stage III	15 (28)
Stage IV	4 (8)
Breast cancer treatments since diagnosis, *n* (%)^a^	
Chemotherapy	48 (88)
Radiation therapy	28 (51)
Surgery	48 (88)
Tamoxifen	11 (20)
Aromatase inhibitors	54 (100)
Number of years on aromatase inhibitors, years mean (SD)	3.4 (1.9)
Time since diagnosis, years mean (SD)	4.5 (2.9)

^a^Participants may have had more than one breast cancer treatment since diagnosis

### Feasibility outcomes of recruitment into the trial

**Fig. 3:**
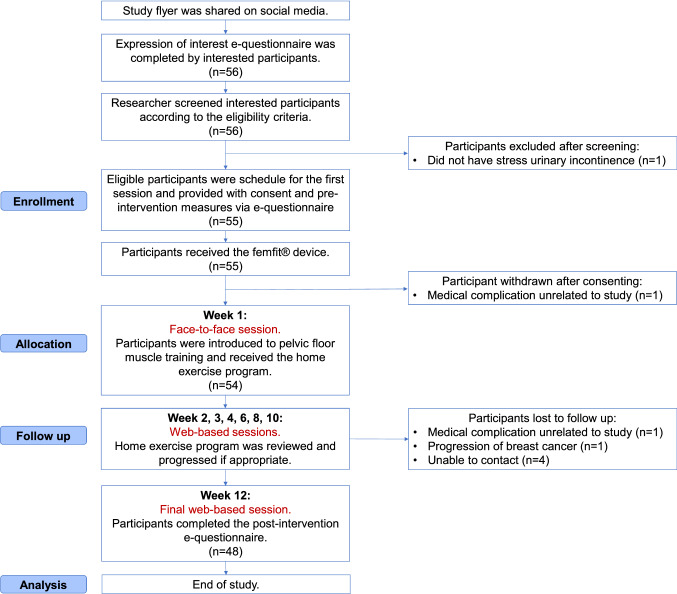
Participant flow through the trial

Figure 3 presents the participant flow through the trial. All women who expressed interest were eligible and subsequently consented to the study (*n* = 55/55). This study had a retention rate of 87% (*n* = 48/55). The mean attendance rate to supervised sessions with the physiotherapist was 95.9% (SD ± 3.1). The mean adherence rate to the home exercise program was 76.3% (SD ± 11.4). Two participants withdrew from the trial due to medical complications unrelated to the study (diagnosis of lung cancer and recurrent urinary tract infections), resulting in a withdrawal rate of 3.6% (*n* = 2/55). There were two adverse events reported throughout the duration of the study (itching in the pubic area and abdominal cramping), both of which were unlikely to have been caused by the use of the femfit^®^ device or performing PFMT.

**Fig. 4:**
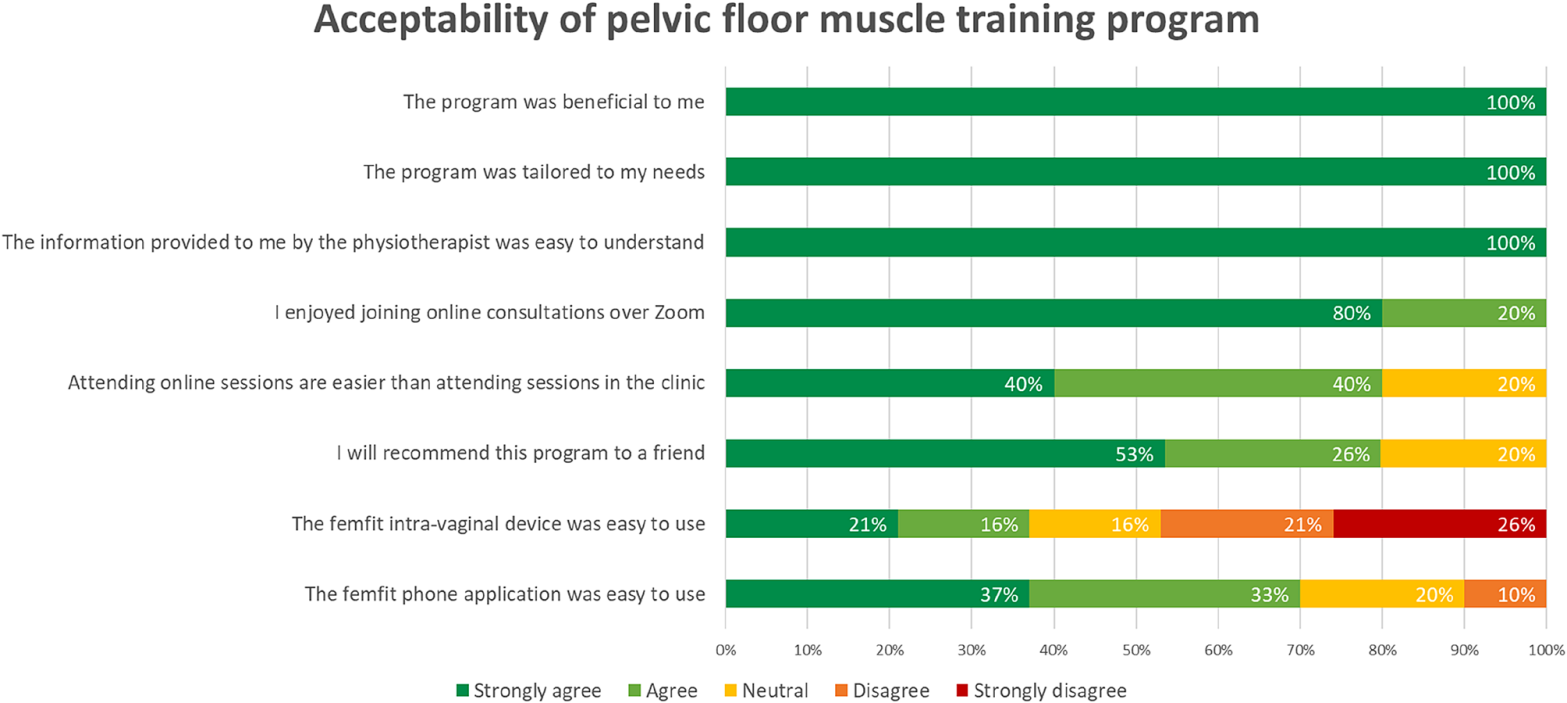
Participant acceptability of the pelvic floor muscle training program

Participant acceptability of the PFMT program is presented in Fig. 4. All participants reported that the program was beneficial, tailored to their needs, provided information and training that was easy to understand. Most participants also agreed that attending physiotherapy sessions online was easier than attending sessions in clinic. However, 47% (*n* = 23/48) of participants reported that the femfit^®^ device was not easy to use. Despite this, 95% of participants (*n* = 46/48) rated that they were either satisfied or very satisfied with the intervention.

### Clinical outcomes from pelvic floor muscle training via telehealth

**Fig. 5:**
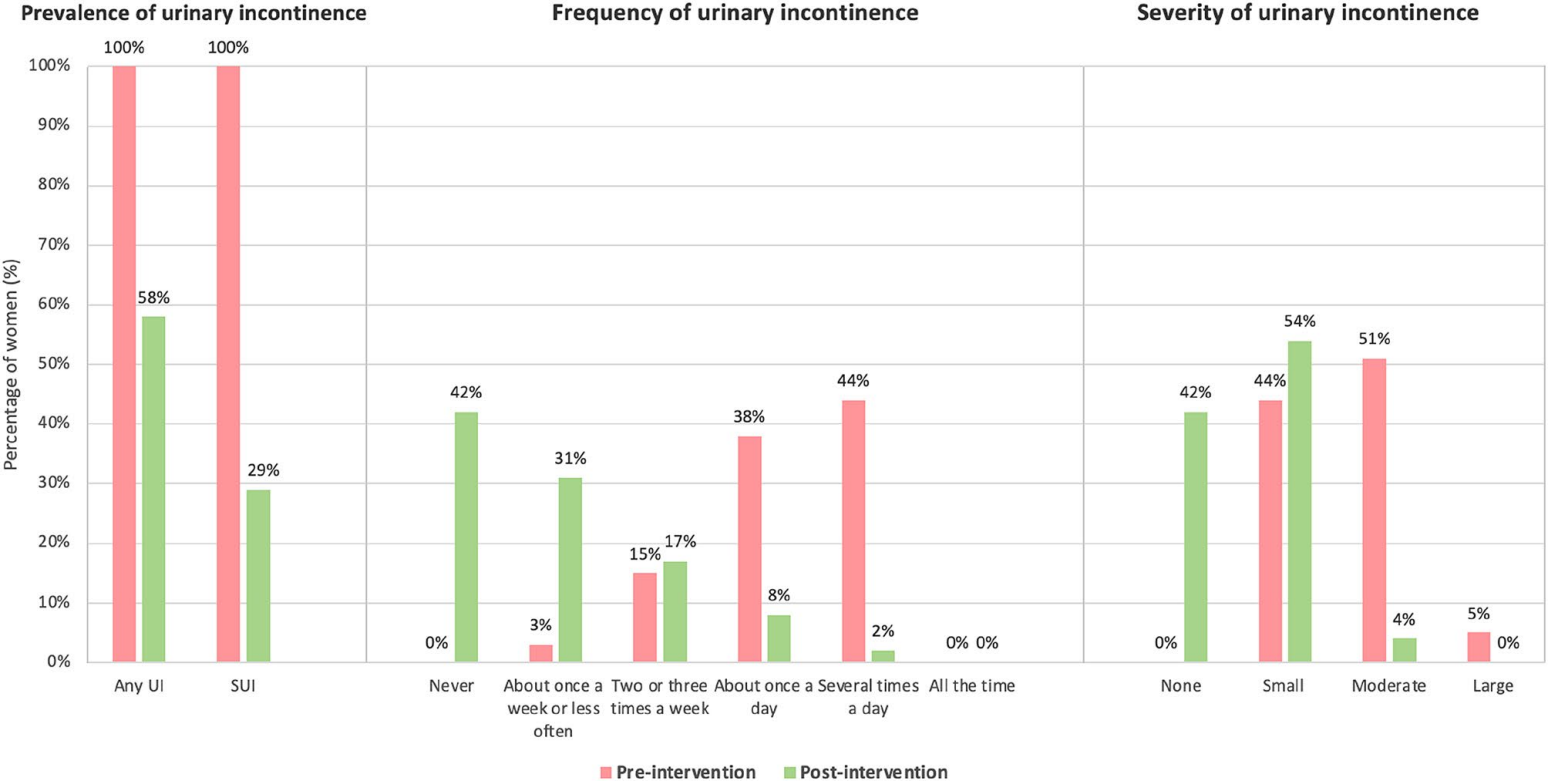
Prevalence, frequency and severity of urinary incontinence pre- and post-intervention. *UI* urinary incontinence, *SUI* stress urinary incontinence

The researchers initially planned for participants to use the biofeedback femfit^®^ device every time they completed the home PFMT. However, due to technical difficulties with the femfit^®^ device which impacted 44/54 (81%) participants, the device was only used during supervised sessions so that the physiotherapist could confirm correct PF muscle contraction technique. The prevalence, frequency and severity of UI assessed prior to and following PFMT intervention are presented in Fig. 5. Results show a statistically significant decline in the prevalence (percentage difference 42%, 95% CI 28, 57%) of UI post intervention.

Table 2 presents clinical outcome measures taken pre- and post-intervention. Participants reported a lower burden of UI post-intervention as indicated by the ICIQ-UI SF score (mean change 9.4, 95% CI 8.5, 10.4). Intravaginal squeeze pressure increased significantly from pre- to post-intervention (mean change 4.8 mmHg, 95% CI 3.9, 5.5). Participants reported a mean 6.6 (95% CI 6.4, 6.8) score on the PGIC after the intervention. Most participants reported that their symptoms were a great deal better and felt that a considerable improvement has been made post-intervention (33/48, 69%).

**Table 2:** Pre- and post-intervention clinical outcomes

Variable	Pre-intervention (*n* = 55)	Post-intervention (*n* = 48)	Mean change (95% CI)^a^
	Mean (95% CI)	Mean (95% CI)	
ICIQ-UI SF score	12.8 (12.1, 13.4)	3.4 (2.3, 4.2)	9.4 (8.5, 10.4)
Frequency of UI	3.3 (3.0, 3.5)	0.9 (0.6, 1.2)	2.4 (2.1, 2.7)
Severity of UI	3.2 (2.9, 3.5)	1.3(0.9, 1.6)	1.9 (1.6, 2.3)
Impact of UI	6.3 (6.0, 6.7)	1.2 (0.8, 1.6)	5.1 (4.7, 5.7)
Intravaginal squeeze pressure, mmHg,	16.5 (14.8, 18.2)	21.3 (19.7, 22.8)	4.8 (3.9, 5.5)
PGIC score		6.6 (6.4, 6.8)	

*UI* urinary incontinence, *ICIQ-UI SF* International Consultation on Incontinence Questionnaire-Urinary Incontinence Short Form, *PGIC* Patients' Global Impression of Change

^a^Only data pre- and post-intervention from *n* = 48 were used in the paired *t* test

## Discussion

This study demonstrated that PFMT delivered via telehealth may be feasible and potentially beneficial in treating any UI, particularly stress UI, in women with breast cancer on AIs. Whilst the high consent rate suggests that PFMT delivered via telehealth was highly feasible, consent rate was calculated based on the number of participants who consented to the study divided by the total number of participants who were eligible but did not consent to the trial. As participants were recruited via advertisement on social media, the true number of eligible participants who saw the advertisement to the study and did not express their interest is unknown. The high consent, attendance and adherence rate indicate that participants who consented to this study were a highly motivated group of women who were interested in engaging with the PFMT intervention.

One point of difficulty with the use of the intravaginal biofeedback device was that several of the eight sensors within the device malfunctioned with frequent use for some users. When a failure occurred, participants were provided with a new device as soon as the fault was detected. It is likely that this resulted in lower adherence to the home exercise program and increased dissatisfaction with the use of the biofeedback device in this study. Despite this, most participants reported satisfaction with the use of the femfit^®^ phone application to guide their home exercise program. These technical difficulties have been investigated by the developers of femfit^®^ and considered in the more recent iterations of the device. With the recent popularity in using at-home devices and phone applications to monitor PFMT [28], it is important that devices are tested in ‘at home’ conditions prior to use by women with UI. It may also be important to have clinician supervision in order to trouble-shoot technical difficulties, and to confirm correct *versus* incorrect contraction technique to the user.

Despite these technological challenges, significant improvements in clinical outcomes were observed. A study investigating the minimal important difference in ICIQ-UI SF score after a randomized clinical trial evaluating efficacy of a supervised 12-week PFMT in women with stress UI reported that a decrease in 2.5 points on the ICIQ-UI SF was clinically meaningful [29]. We observed a much higher reduction in ICIQ-UI SF score (nine points) post PFMT, which suggests the change may have been clinically meaningful for these participants, and concurs with our results from the PGIC measure. Another study assessing PF muscle strength using manometry measures following PFMT in women without cancer reported a mean difference of 5.04 cmH_2_O (approx. 3.8 mmHg) between the PFMT and control groups [30]. Despite participants in our study having weaker PF muscles pre-intervention compared to incontinent women without cancer as reported in the previous study, we saw a greater improvement in PF muscle strength (4.8 mmHg) following our intervention. Further studies including randomized controlled trials that are powered to detect a significant difference in the prevalence of UI and a change in PF muscle strength are required to confirm these results.

### Limitations

There are several limitations that need to be considered. Firstly, as the physiotherapist was unable to visualize the participant’s lower limbs via telehealth, any co-contraction of gluteal and hip adductor muscles would not have been corrected. Researchers were therefore unable to confirm the presence of accessory muscle activation. Secondly, when a femfit^®^ device showed signs of technical malfunction, a replacement device was issued. Due to the high number of malfunctions experienced throughout the study, most participants used a different femfit^®^ device to measure PF muscle strength pre- and post-intervention. While no data on inter-device reliability has been published, rigorous testing has been undertaken by developers to comply with regulatory standards such as the Australian Therapeutic Goods Administration [12]. As the one physiotherapist who implemented the PFMT program received training prior to starting the trial, to achieve a level of competency to match standard clinical practice in our state, the generalizability of feasibility outcomes (such as program satisfaction and acceptability) to physiotherapists who have not received this training cannot be assumed. However, any variation in care that may occur with two or more supervising clinicians can be minimized if clinicians are adequately trained in the delivery of PFMT using the femfit^®^ device and standardized protocols are followed. Thirdly, as PF muscle strength measurements were extracted as a part of the PFMT program on the femfit^®^ phone application, participants were asked to squeeze for differing durations pre- and post-intervention (week 1: six second holds; week 12: ten second holds). Due to this, there may have been an element of fatigue with the longer hold which may have resulted in an underestimation of maximal intra-vaginal squeeze pressure in the post interventional measure. Lastly, results of the feasibility measures (such as high consent, attendance and adherence rates) may not be accurate measures due to selection bias where highly motivated women expressed their interest in participating in this study over social media. We also noted a large difference in the estimated consent rate used to base our sample size calculation on (15%), and the consent rate observed in this study (100%). This is likely because the estimated consent rate was based on a clinic-based recruitment strategy while our study employed an online social media recruitment. The consent rate in clinic is likely to be lower [31], and should be considered in future studies recruiting from clinical settings. Despite these limitations, we observed high attendance and adherence rates, good acceptability and satisfaction of the program and no adverse reactions linked to the study, indicating that PFMT delivered via telehealth may be feasible, safe and potentially beneficial in treating UI in women with breast cancer.
